# Effects of an Existential Nursing Intervention for College Students in the COVID-19 Pandemic Situation

**DOI:** 10.3390/ijerph18105268

**Published:** 2021-05-15

**Authors:** Sunhee Cho, Sun Joo Jang

**Affiliations:** 1 Department of Nursing, Mokpo National University, Muan 58554, Korea; scho@mokpo.ac.kr; 2 Red Cross College of Nursing, Chung-Ang University, Seoul 06974, Korea

**Keywords:** logotherapy, stress, depression, students

## Abstract

This study designed an intervention based on logo-autobiography to reduce Korean college students’ stress and depression and help them find meaning in their lives amidst the COVID-19 pandemic. A quasi-experimental design was used to conduct group interventions among college students. A total of 22 and 26 participants were included in the experimental and control groups, respectively. The experimental group received six sessions of a logo-autobiography for college students (LAC). The effects of the LAC interventions were assessed at baseline, post-intervention, and four weeks after the program’s end to determine their retention rate. The effects of group, time, and the group-by-time interaction were verified using generalized estimating equations with an autoregressive correlation structure. The experimental group exhibited significantly lower levels of stress and depression and higher levels concerning the meaning of life than the control group. However, only the effects on stress and the meaning of life continued four weeks after the intervention’s end. Based on this study’s results, LAC can be considered a useful method for reducing stress and depression in college students who have just started their adult life, as well as for aiding them in their pursuit for the meaning of life.

## 1. Introduction

Due to the stressful nature of modern society, both social and economic aspects can cause life stress in college students entering adulthood [1,2,3]; furthermore, the overall seriousness of modern life stress is increasing [4]. In addition to academic and employment stress, students are also constantly worried about being judged and evaluated by others, which is detrimental to individual happiness and may cause mental health issues [5]. Students suddenly must adapt to a new life where they are no longer under the control or care of their parents and high schools. Instead, they must transition into early adulthood and resolve numerous issues (i.e., economic problems), which may lead to rapid increases of their stress levels [6].

Moreover, due to the coronavirus disease-19 (COVID-19) pandemic, most colleges in 2020 offered their semester classes online. Consequently, students were subjected to social distancing and restricted social activities, which resulted in isolation and stress [7,8]. Due to the COVID-19 pandemic, college students have been facing mental health problems stemming from negative emotions [9], including anxiety, fear, a decreased tolerance for uncertainty, and insecurities in public [10,11,12]. This has happened to such an extent that the term “corona blues”—referring to the depression and lethargy caused by self-isolation and social distancing—has emerged [13]. This suggests that the mental health status of college students has significantly worsened during the COVID-19 pandemic [14].

Previous studies reported that mental health issues in college students may increase their risk of suicide [15,16] and lead to various addiction problems; especially since losing their meaning of life can lead to a vicious cycle of threatened mental health [17]. However, depending on their subjective perspectives, stress can have different effects on individuals. For example, factors related to the “meaning of life,” which seek meaning in pain, act as important protective factors in groups with vulnerable mental health [18,19]. Milman et al. [20] found that discovering meaning has mediated social isolation and mitigated coronavirus anxiety during the pandemic. In Negri et al.’s study [21], an expressive-writing intervention facilitated reflecting on and reorganizing the personal meaning of negative events and elaborating emotions during COVID-19 confinement. Logotherapy, also known as meaning-centered psychotherapy, is a psychological intervention proposed by the developer Viktor Frankl [22] that aims to help individuals discover the meaning of life through creative, experimental, and attitude values based on existential psychotherapy. Therefore, logotherapy-based interventions are considered useful in crisis situations, such as the COVID-19 pandemic, for improving and preventing mental health problems in college students.

### Background

Frankl [23] coined the term “meaning of life” as a variable that refers to the tendency of human nature to pursue the meaning of life, and that failing to discover this meaning would lead to feelings of emptiness and depression. Similarly, previous studies observed that the meaning of life variable was positively correlated with mental health [6,18], life satisfaction [24], and hope [25], and negatively correlated with suicidal thoughts [26] and depression [25]. Additionally, the meaning of life variable is a predictor of psychological well-being [27] and post-traumatic growth [15]. Furthermore, this variable mediates the effects of stress on depression [25], and its low and high scores are predictive of suicidal ideation and hope, respectively [19]. Therefore, interventions that can improve the meaning of life variable in college students are highly useful for preventing mental health problems, such as stress, depression, and suicidal thoughts.

Recently, many mental health professionals started implementing logotherapy to perform individual or group interventions and, subsequently, the number of studies verifying its effects are increasing [28,29]. Several of these studies developed protocols and conducted experimental studies with standardized interventions in accordance with developed protocols to scientifically verify their effects. These studies include Wong’s [30] meaning-centered therapy, a meaning-centered psychotherapy for cancer patients and their families [31], and a logo-autobiography (LA) that combined logotherapy and autobiography writing methods [32,33]. Traditional logotherapy is based on “talking therapy,” which involves conversations between the therapist and the subject, whereas LA, which combines autobiography writing and logotherapy, benefits from life reviews and expressive writing in combination with logotherapy. Studies have discovered that LA can be effective in reducing stress [8,34], improving the mental health of wives of men with alcoholism [35], alleviating depression [29,32,33] and improving the ability to cope with depression among Korean immigrant women in the United States [36], and enhancing meaning in life [37,38].

College students, who are in early adulthood, face the challenging tasks of career planning and job preparation [26]. Living in isolation during the COVID-19 era, they could experience stress and depression, potentially losing their sense of purpose [7,8,9]. This raises the need for existential reflection on who they are. Thus, a logo-autobiography for college students (LAC) protocol was developed [39] for students who experienced stress and depression during the COVID-19 pandemic [10]. LAC is a group psychological intervention that helps college students to assume a different perspective from which they can re-interpret their pain and alter their attitudes toward suffering by helping them discover the purpose and meaning of life. LAC is based on the principles of logotherapy and the LA protocols developed for immigrant women with depression [32]. Both LA and LAC involve the therapy of autobiographical writing in which common topics are explored, group members share their writings, exchange feedback, and discover meaning in their lives. The difference between the two methods lies in the latter’s emphasis on the uniqueness and purpose of life as a college student [39]. Therefore, this study assessed the effects of LAC as an intervention strategy targeted at improving the mental health of, and preventing mental problems in, college students during the COVID-19 pandemic. This experimental study validated LAC as a useful intervention for the mental health management of college students during the COVID-19 pandemic as well as presented basic data concerning college students to assist persons worldwide suffering during the COVID-19 pandemic. The following hypotheses were proposed:

**Hypothesis** **1** **(H1).** *The experimental group that participated in LAC would exhibit a lower score for perceived stress than the control group after the intervention.*

**Hypothesis** **2** **(H2).** *The experimental group that participated in LAC would exhibit a lower score for depression than the control group after the intervention.*

**Hypothesis** **3** **(H3).** *The experimental group that participated in the LAC would exhibit a higher score for the meaning of life variable than the control group after the intervention.*

## 2. Materials and Methods

### 2.1. Participants

This study was conducted with 52 Korean students from four different colleges in two cities, who were all enrolled in four-year academic programs. A minimum sample size of 44 was calculated using the G*Power 3.1.9.7 for an F test with a two-way repeated measures analysis of variance, effect size 0.25, power 0.95, and three measurements (i.e., pre, post, and four weeks after intervention). A total of 26 participants were recruited for both the experimental and control groups after accounting for possible dropouts. The inclusion criteria were students registered for the semester who were able to communicate, read, and answer a survey by themselves, who were willing to participate after being informed of the study purpose. The exclusion criteria were students on leave, or who experienced difficulties in communicating, reading, and writing, or were unwilling to participate in the study. Four participants from the experimental group dropped out immediately after their enrolment due to their individual schedules; thus, 22 (response rate 84.6%) and 26 (response rate 100%) participants were included in the experimental and control groups, respectively.

### 2.2. Design

A non-randomized controlled study design was used incorporated, with a pre-test, post-test, and follow-up.

### 2.3. Data Collection and Intervention

Data were collected between July and December 2020, after receiving approval from the university’s institutional review board. We recruited participants for the experimental group by posting flyers at a university, different departments’ homepages, and on students’ social media pages. In order to counteract the diffusion effect, the control group’s participants were recruited from four different universities in a similar manner.

An intervention strategy based on logotherapy and LA was developed by Cho and Do [39] to suit the characteristics of college students during their developmental stages. This strategy was called LAC and its validity, applicability, and usefulness were assessed. Two logotherapy experts certified by the Viktor Frankl Institute of Logotherapy, including the researcher (S.C.), conducted these LAC sessions. The researcher, who was involved in the development of LA, on which LAC is based, is a psychiatric/mental health nurse practitioner in New York State and a mental health nursing professor in Korea. Participants in the experimental group received six 90-min LAC sessions once a week, for six weeks. Furthermore, these sessions included between two and four participants from the experimental group in a seminar room of a university. The main contents of each LAC session are described in Table 1.

**Figure 1:**
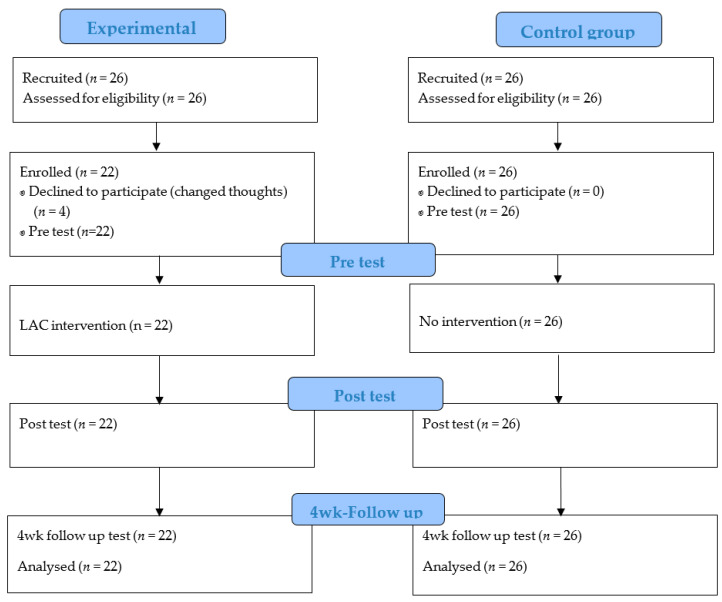
Flow chart of the participants’ recruitment and participation.

Each session has four stages: (a) warm-up for 15 min; (b) writing one’s autobiography on each topic for 30 min; (c) presenting and sharing the written autobiography with the group for 30 min; (d) sharing one’s feelings and providing feedback for 15 min. In 3–6 sessions focused on finding meaning in life, we dealt with participants’ experiences during the COVID-19 pandemic as important life events. With effort, the protocol was standardized, and a structured program was conducted through regular meetings of therapists. For the control group, the researchers conducted a pre-test, post-test, and follow-up test without conducting the interventions (Figure 1).

### 2.4. Measures

#### 2.4.1. Perceived Stress

The Perceived Stress Scale (PSS) was developed by Cohen et al. [40] and is one of the most commonly used instruments to assess stress. It is a self-report 10-item instrument that uses a Likert scale to measure the intensity of participants’ perceived stress and the stress of daily life [40]. Scores range from 0 to 40; a higher PSS score indicates higher levels of stress. Reliability was assessed using Cronbach’s α for the PSS, which ranged from 0.84 to 0.86 at the time of development [40] and had a value of 0.87 in this study.

#### 2.4.2. Depressive Symptoms

Depressive symptoms were measured using 10 items from the Center for Epidemiologic Studies Depression Scale (CES-D-10) [41], which was developed to screen for depressive symptoms. Participants answer “yes” or “no” to items describing feelings and actions experienced in the previous week. Negative items exist that are reverse scored. Higher scores indicate greater depressive symptoms. Scores range between 0 and 10. Cronbach’s α value for the CES-D-10 [41] was 0.80 at the time of development and 0.81 in this study.

#### 2.4.3. Meaning of Life

Participants’ meaning of life variables were measured using the Korean version of the Purpose-In-Life (PIL-K) questionnaire, which was developed by Crumbaugh and Maholick [42] and culturally adapted by Park and Lee [43]. It was developed to operationalize Frankl’s concept of an existential vacuum. The PIL-K is a 20-item scale known to exhibit high reliability (Cronbach’s α of the original scale = 0.86; Cronbach’s α in this study = 0.97). Each item is rated on a seven-point scale from 1 to 7; total scores range from 20 (low meaning) to 140 (high meaning). A score of 112 or higher on the PIL-K scale suggests that individuals derive a sense of meaning in life, namely holding the belief that their lives are generally meaningful. Cronbach’s α value for the culturally validated PIL-K [43] was 0.91 at time of adaptation, while it was 0.93 in our study.

### 2.5. Ethical Considerations

The study was conducted according to the guidelines of the Declaration of Helsinki and approved by the Institutional Review Board of Mokpo National University (Approval No. MNUIRB-20190904-SB-014-01; Approval Date: 31 December 2019). The study’s purpose, methods, and guarantee of anonymity were explained to the participants before obtaining their consent for participation in the study. Furthermore, the participants were also informed that they could withdraw from the study at any time they wished; anonymous return envelopes were used to ensure anonymity. The investigation was carried out in accordance with the latest version of the Declaration of Helsinki.

### 2.6. Data Analysis

The data were analyzed using SPSS 24 (SPSS Inc., Chicago, IL, USA). Descriptive statistics were used to analyze the participants’ general characteristics, and both a t-test and a chi-squared test were performed to verify the homogeneity of the control and experimental groups. The effects of group, time, and group-by-time interactions between the groups were verified using generalized estimating equations (GEEs) with an autoregressive correlation structure. Additionally, participants’ general characteristics were controlled. All participants were assigned to the same group throughout the study, and a per-protocol analysis intention was applied.

## 3. Results

### 3.1. Homogeneity Test of Participants’ General Characteristics and Dependent Variables

Table 2 displays the test results concerning the homogeneity of the participants’ general characteristics and dependent variables. The mean age of the participants was 21.54 years (SD 2.84), and 72.9% of the participants were female. Furthermore, 62.6% of the students majored in either science, technology, engineering, or mathematics, while 31.3% of the students’ socioeconomic status was middle class. Approximately 75% of the participants exhibited moderate academic performance (i.e., a grade point average of 3.0–4.0). All variables, except for the participants’ subject majors, were homogeneous between groups (Table 2).

### 3.2. Verification of Logotherapy Effects

**Figure 2:**
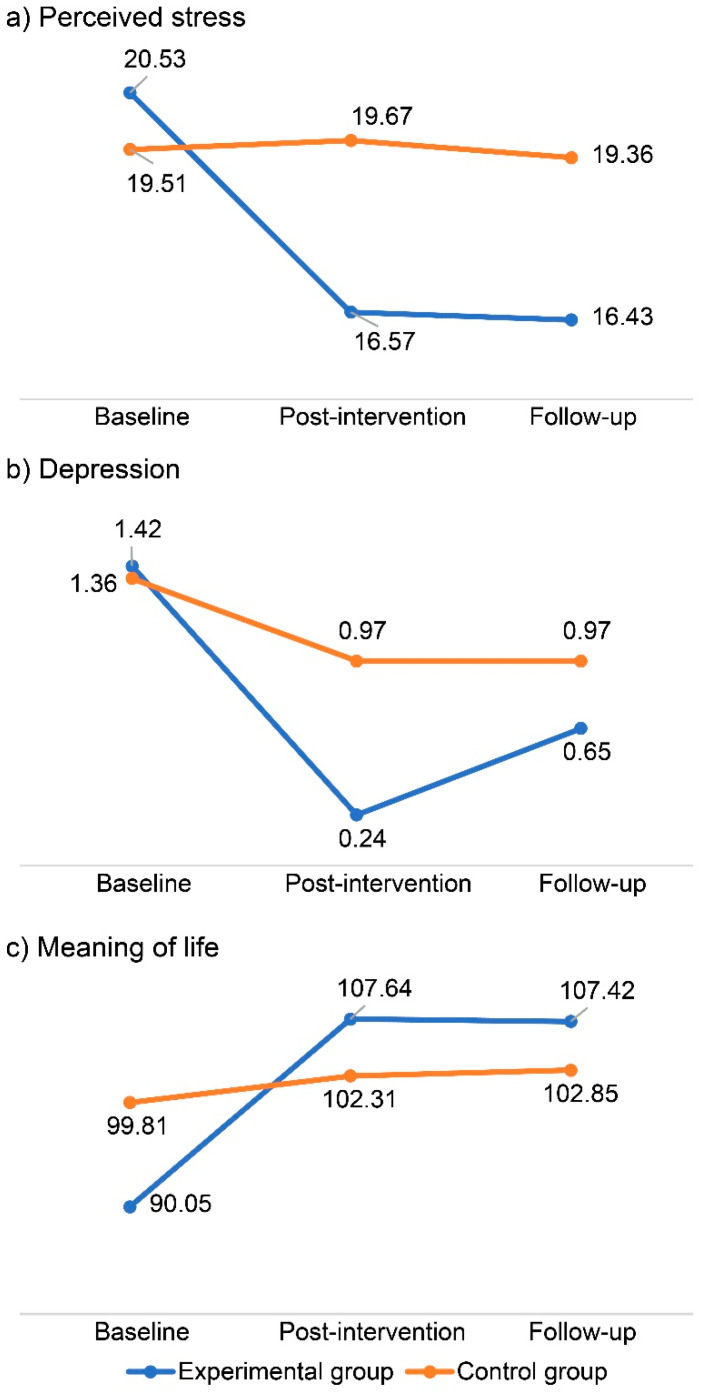
Outcome changes in group comparison; effect of the LAC on (**a**) perceived stress, (**b**) depression, and (**c**) meaning of life. The estimated mean is represented.

The effects of logotherapy on stress, depression, and meaning of life, as verified by the GEE, are explored below. Furthermore, changes in stress, depression, and meaning of life at baseline, post-intervention, and follow-up are shown in Figure 2. A comparison of the changes in dependent variables between the two groups over time are displayed in detail in Table 3.

#### 3.2.1. Stress

Hypothesis 1 was supported, namely the experimental group that participated in LAC exhibited a lower score for perceived stress than the control group after the intervention. The results of the GEE analysis are shown in Table 3; the main effects of group and time were not significant. However, a significant group-by-time interaction was discovered (post-intervention Wald’s test = 8.86, *p* = 0.003; follow-up Wald’s test = 13.65, *p* < 0.001). Furthermore, the analysis indicated that, after adjusting for demographic covariates, the stress of the experimental group decreased significantly more than that of the control group (Table 3).

#### 3.2.2. Depression

Hypothesis 2 was supported, namely the experimental group that participated in LAC exhibited a lower score for depression than the control group after the intervention. Although the main effects of group and time were not significant, a significant group-by-time interaction was discovered (post-intervention Wald’s test = 4.35, *p* = 0.037). However, there was no significant group-by-time interaction during follow-up, which was assessed to observe the retention of effects after the program’s end (Wald’s test = 0.53, *p* = 0.465). Furthermore, the analysis indicated that, after adjusting for demographic covariates, the depression of the experimental group decreased significantly more than that of the control group (comparison between baseline and post-intervention; Table 3).

#### 3.2.3. Meaning of Life

Hypothesis 3 was supported, namely the experimental group that participated in the LAC exhibited a higher score for meaning of life than the control group after the intervention. Once again, the main effects of group and time were not significant. However, a significant group-by-time interaction was discovered (Wald’s test = 23.65, *p* < 0.001; follow-up Wald’s test = 13.19, *p* < 0.001). The analysis indicated that, after adjusting for demographic covariates, the meaning of life scores of the experimental group increased significantly more than those of the control group (Table 3).

## 4. Discussion

This quasi-experimental research study verified the effects of LAC on the stress, depression, and meaning of life of Korean college students. The results of this study clarified the effects of LAC, semantic writing therapies, various types of logotherapy, and other aspects associated with logotherapy on college students in the pandemic era.

In this study, we observed that the experimental group, which participated in LAC, exhibited less stress and depression than the control group. Prior research on LA [31,32,33,35,37] has emphasized reinterpreting the meaning of suffering in patients, middle-aged individuals, and people who have experienced trauma. Given college students’ need for identity recognition, LAC focuses on the existential question of “what kind of being am I?” Furthermore, college students who participated in non-face-to-face classes during the COVID-19 pandemic exhibited very high levels of academic and life stress, similar to the stress levels associated with disaster experiences. A study reported that logotherapy helped counteract college students’ stress, which rapidly increased during the lockdown period [8]. Furthermore, several other studies have also discovered positive effects on college students’ depression by implementing logotherapy. One study of students with problematic drinking behaviors reported that both meaning of life and depressive symptoms improved after six sessions of logotherapy [44]. In another study, students’ addiction potential decreased while their psychological well-being increased [45]. Therefore, since the addiction potential and psychological well-being of college students were altered through logotherapy, it may have long term positive effects on their drug- or alcohol-related behaviors. In another study, 10 sessions of logotherapy led to significant improvements in students’ depressive symptoms, and these effects were retained up to four weeks after the study period ended [37]. This finding reinforces our results that LAC improved the depression of Korean college students. However, unlike the results of Robatmili et al. [37], the improved depression score was not maintained in the follow-up test four weeks after our study. Nevertheless, the increased levels of social distancing resulting from high amounts of confirmed COVID-19 cases, which overlapped with the four-week follow-up period, may have affected the depression levels of college students. A recent study confirmed that depression and anxiety are prevalent among college students during the COVID-19 pandemic [46,47,48]. In previous studies [21,49], meaning making mediated pandemic depression while expressive writing positively influenced emotion. As Reverté-Villarroya et al. [14] emphasized, there is a desperate need for interventions that manage the negative emotions of college students, and LAC is regarded as a viable intervention strategy. Therefore, we suggest that future studies develop a platform and an app [50] that provides online intervention programs to multiple users at once, to promote continuous, autonomous, and ubiquitous participation.

In our study, the experimental group, which participated in LAC, exhibited more improvements in their scores of meaning of life than the control group; these effects were retained until four weeks after the program’s end. In a large-scale descriptive longitudinal study involving college students [18], the students’ discovered meaning in their lives, which affected their pursuit of the meaning of life for up to six months later. Furthermore, when considered longitudinally, this pattern persisted for up to a further six months, while the discovery of meaning also increased with time. Additionally, the college students who discovered more meaning in their lives also exhibited more positive mental health characteristics. This suggests that the activity of discovering the meaning of life causes effects that improve and sustain college students’ mental health. Furthermore, Robatmili et al. [37] indicated that logotherapy significantly improved the meaning of life in college students, and that these improvements persisted until four weeks after the program’s end. Similar results of logotherapy on the meaning of life are reflected in both a review by [34] and a study by Mason and Nel [51] concerning a meaning-oriented life. Additionally, these results are consistent with studies of older persons [52] and patients with paralysis [38].

Min et al. [53] reported that a high meaning of life significantly increased the stress resilience of patients diagnosed with mental disorders related to depression and anxiety; furthermore, Southwick et al. [34] stated that logotherapy improved stress resilience. These results suggest that logotherapy may improve participants’ stress resilience by improving their perceived meaning of life. To summarize, increasing the perceived meaning of life is effective at managing the stress-related problems of college students during the pandemic period, and it is necessary to provide various logotherapy interventions, such as LAC. Previously, Nash [54] reasoned that professors should assist college students in discovering the meaning of life at a university level. Since the mental health of college students is now threatened during the COVID-19 pandemic [11], universities and professors must be proactive.

Of the various psychological interventions that use logotherapy, both LA and LAC particularly involve writing. In a study by Cho [35], the wives of persons with alcoholism in Korea were asked to write autobiographies according to their creative, experiential, and attitudinal values and share the contents during feedback sessions. The study found that through the intervention, participants realized and accepted the source of their pain, and discovered meaning in their lives. Additionally, the stress caused by trauma related to their husbands’ drinking behaviors decreased; these effects persisted until four weeks after the intervention. Similarly, LA interventions also significantly improved the depression and meaning of life variables of Korean immigrant women in the U.S. These effects also lasted until four weeks after the interventions [32,33]. Furthermore, an enhanced LA improved the depression, meaning of life, and coping skills of Korean immigrant women with depression in the U.S., through added coping skills [36]. The LA was based on autobiographical writing in all these studies. Thus, since participants were able to effectively interpret past trauma in cognitive ways, deeper reviews of their lives were possible than would have been achieved with psychological interventions that only use spoken words.

We posit that the LAC in our study had similar effects as autobiographical writing; however, subsequent studies are needed to verify the effects of autobiographical writing by applying various psychological interventions. Furthermore, since LA was developed for middle-aged and older adults, it primarily focuses on participant’s past trauma and finding the meaning in their pain through autobiographical writing [32,33,36]. However, the LAC provided in this study was intended for college students, and thus, differs from LA in that it focused on present trauma, a desire to find identity, and anxiety toward the future. It is important to understand the characteristics of the different developmental stages of college students and select topics that satisfy their existential needs.

### Limitations and Future Directions

There are certain limitations of this study that need to be addressed. This study was conducted with college students from four different universities in two Korean cities and was not a randomized controlled trial. Therefore, selection bias may have occurred, and the extent to which the results may be generalized is limited. Future studies should address these limitations through the use of randomized controlled trials and longitudinal studies that assess the effects of LAC on finding meaning in life and maintaining the mental health of college students. Moreover, we suggest a three-group experimental study to compare the effects between different types of logotherapy or other expressive-writing protocols.

## 5. Conclusions

By verifying the effects of LAC on stress, depression, and meaning of life, this study provided the basic data required to improve the mental health of college students. Furthermore, it discovered that these effects persisted until four weeks after the interventions’ end. These findings suggest that this program may be an effective intervention strategy for college students to improve their mental health, prevent mental health problems, and help them find the meaning of life from existential emptiness. The findings of this study revealed that LAC can reduce patients’ stress and depression and improve their meaning of life during the disastrous COVID-19 pandemic. Furthermore, LAC might be able to prevent college students from dropping out and can be utilized as a suicide prevention strategy in schools by increasing students’ meaning of life, and thus, improving their mental health.

## Figures and Tables

**Table 1:** Topic, goal, activity, and value in LAC ^1^.

Session	Topic	Goal	Activity	Value
1	Self-introduction	Introducing and thinking of oneself	Writing of one’s own symbol or nickname	Creative, experiential, attitudinal
2	Exploring the unique self	Recognizing my uniqueness	“Am I unique?” Writing of my purpose in life, desires, or interests	Creative, experiential, attitudinal
3	Encounters in my stories	Finding meaning through life reviews	Drawing my life graph and writing about experiences	Attitudinal
4	My crises and choices	Finding meaning in times of crisis	Writing about my crises	Attitudinal
5	Self-transcendence in my stories	Finding meaning in self-transcendence	Writing about experiencing self-transcendence	Attitudinal
6	Making a tree of the meaning in life	Identifying discovered meanings	Filling in blank meaning tree	Creative, experiential, attitudinal

^1^ LAC: Logo-autobiography for college students.

**Table 2:** Homogeneity test of participants’ characteristics and dependent variables (*n* = 48).

Characteristics	Categories	Exp ^1^ (*n* = 22) *n* (%) Mean (SD)	Cont ^2^ (*n* = 26) *n* (%) Mean (SD)	Total (*n* = 48) *n* (%) Mean (SD)	χ^2^ or *t*	*p*
Gender	Male	5 (22.7)	8 (30.8)	13 (27.1)	0.39	0.746
	Female	17 (77.3)	18 (69.2)	35 (72.9)		
Age (years)		20.86 (1.49)	22.12 (3.54)	21.54 (2.84)	1.55	0.129
Major	Humanities, arts and social sciences	13 (59.1)	5 (19.2)	18 (37.5)	8.08	0.007 *
	STEM ^3^	9 (40.9)	21 (80.8)	30 (62.6)		
SES ^4^	High	1 (4.5)	1 (3.8)	2 (4.2)	0.94 ^†^	0.815
	Middle	7 (31.8)	8 (30.8)	15 (31.3)		
	Low	5 (22.7)	9 (34.6)	14 (29.2)		
	Not-know	9 (40.9)	8 (30.8)	17 (35.4)		
Academic performance (GPA ^5^ 4.5)	High (≥4.0)	3 (13.6)	7 (26.9)	10 (20.8)	3.40 ^†^	0.183
	Middle (≥3.0)	17 (77.3)	19 (73.1)	36 (75.0)		
	Low (<3)	2 (9.1)	0 (0)	2 (4.2)		
Stress		20.64 (4.77)	19.00 (6.15)	19.75 (5.56)	−1.02	0.315
Depression		2.05 (1.79)	2.15 (2.28)	2.10 (2.05)	0.18	0.857
Meaning of life		85.36 (19.48)	93.58 (18.28)	89.81 (19.09)	1.51	0.139

^1^ Exp: experimental group; ^2^ Cont: control group; ^3^ STEM: science, technology, engineering, and mathematics; ^4^ SES: socioeconomic status; ^5^ GPA: grade point average; * *p* < 0.05; ^†^ Fisher’s exact test.

**Table 3:** Effects of logotherapy on participants’ stress, depression, and meaning of life (*n* = 48).

95% Wald CI						
Variables	*B*	SE	Lower	Upper	Wald χ^2^	*p*
**Stress**						
Group ^†^						
Exp	0.47	1.51	−2.49	3.43	0.10	0.754
Time ^†^						
Baseline	0	0				
Post-intervention	0.15	0.75	−1.32	1.63	0.04	0.838
Follow-up	−0.15	0.59	−1.31	1.00	0.07	0.793
Interaction of groups and time ^†^						
Baseline	0	0				
Post-intervention	−4.12	1.38	−6.81	−1.40	8.86	0.003 **
Follow-up	−3.94	1.08	−6.06	−1.81	13.19	<0.001 **
**Depression**						
Group ^†^						
Exp	0.35	0.46	−0.55	1.25	0.58	0.445
Time ^†^						
Baseline	0	0				
Post-intervention	−0.19	0.16	−0.51	0.11	1.55	0.214
Follow-up	−0.19	0.19	−0.57	0.17	1.10	0.295
Interaction of groups and time ^†^						
Baseline	0	0				
Post-intervention	−0.28	0.32	−1.29	−0.04	4.35	0.037 *
Follow-up	−0.67	0.38	−1.02	0.47	0.53	0.465
**Meaning of life**						
Group ^†^						
Exp	−7.26	5.18	−17.42	2.89	1.97	0.161
Time ^†^						
Baseline	0	0				
Post-intervention	2.50	1.71	−0.86	5.86	2.13	0.144
Follow-up	3.04	2.01	−0.90	6.98	2.29	0.130
Interaction of groups and time ^†^						
Baseline	0	0				
Post-intervention	15.09	3.10	9.01	21.17	23.65	<0.001 **
Follow-up	14.33	3.88	6.73	21.93	13.65	<0.001 **

^1^ Exp: Experimental group, covariates: age, major, academic performance. *p* value: GEE model adjusted for covariates. * *p* < 0.05, ** *p* < 0.01. ^†^ Reference: control group for group effect; baseline values for time effect; and baseline values of control group for interactions.

## Data Availability

The data presented in this study are available on request from the corresponding author and with permission of the Institutional Review Board of M University.
